# Sensory Attenuation in Sport and Rehabilitation: Perspective from Research in Parkinson’s Disease

**DOI:** 10.3390/brainsci11050580

**Published:** 2021-04-30

**Authors:** Joshua Kearney, John-Stuart Brittain

**Affiliations:** 1 School of Psychology, University of Birmingham, Edgbaston, Birmingham B15 2TT, UK; 2 Centre for Human Brain Health, University of Birmingham, Edgbaston, Birmingham B15 2TT, UK; j.brittain@bham.ac.uk

**Keywords:** sensory attenuation, Parkinson’s disease, basal ganglia, motor control, rehabilitation

## Abstract

People with Parkinson’s disease (PD) experience motor symptoms that are affected by sensory information in the environment. Sensory attenuation describes the modulation of sensory input caused by motor intent. This appears to be altered in PD and may index important sensorimotor processes underpinning PD symptoms. We review recent findings investigating sensory attenuation and reconcile seemingly disparate results with an emphasis on task-relevance in the modulation of sensory input. Sensory attenuation paradigms, across different sensory modalities, capture how two identical stimuli can elicit markedly different perceptual experiences depending on our predictions of the event, but also the context in which the event occurs. In particular, it appears as though contextual information may be used to suppress or facilitate a response to a stimulus on the basis of task-relevance. We support this viewpoint by considering the role of the basal ganglia in task-relevant sensory filtering and the use of contextual signals in complex environments to shape action and perception. This perspective highlights the dual effect of basal ganglia dysfunction in PD, whereby a reduced capacity to filter task-relevant signals harms the ability to integrate contextual cues, just when such cues are required to effectively navigate and interact with our environment. Finally, we suggest how this framework might be used to establish principles for effective rehabilitation in the treatment of PD.

## 1. Introduction

People with Parkinson’s Disease (PD) face debilitating symptoms that often begin years before disease diagnosis [1], worsen over time and significantly affect quality of life. Dopamine replacement therapy is used to treat PD and can be effective for long periods [2], though it does not improve all cardinal symptoms of this neurodegenerative disorder [3], with high levels of wearing off over time and considerable dissatisfaction among patients [2]. While techniques such as deep brain stimulation can be employed with relative success [4], they are still associated with significant shortfalls [5], not least of which are the narrow inclusion criteria deemed necessary for high efficacy [6], and the significant investment in time required for effective stimulator titration. This means there is a need for effective therapies that can be utilised in prodromal and early-stage PD in a preventative capacity, which may delay, or even complement later stage medical or surgical options. Various exercise therapies have been developed which have involved the manipulation of sensory feedback [7,8], manipulation of movement amplitude [9], multi-sensory cueing strategies [10,11,12,13,14], action-observation and motor imagery [15], resistance training [16], forced-exercise protocols [17,18,19,20] and dance [21,22]. Whilst varying degrees of success have been reported, an optimal strategy has not been identified [14] and underpinning mechanistic principles for effective rehabilitation remain elusive.

PD results from gross degeneration of midbrain dopaminergic nuclei which innervate the basal ganglia (BG), leading to abnormal patterns of activity in BG pathways. However, it is not so straightforward how such abnormal patterns of activity give rise to the wide-ranging motor and non-motor symptoms of PD, and yet it is important in the development of effective treatments. Here, we first investigate the phenomenon of sensory attenuation which traditionally describes the suppression of sensory input resulting from motor intent. We re-examine the literature with an explicit consideration of Task-Relevance and reframe sensory attenuation as the distinct context-dependent perception of two identical stimuli. We propose that this novel framing reconciles a previously contradictory literature whilst still incorporating the concept of movement modulating sensory input. Such reframing presents sensory attenuation paradigms as a useful means to gain insight into how people monitor task-relevant signals and utilise contextual cues during movement, a concept not well captured by current sensory attenuation theory.

Next, we explore the neural substrates of sensory attenuation and their somewhat paradoxical conjunctions with Parkinson’s disease, which implicate the BG. Non-motor symptoms of PD are increasingly gaining recognition as an intrinsic part of the disease [23,24,25]. This is supported by the recognition that BG circuits contribute to a plethora of non-motor as well as motor functions in a sensory capacity [26,27,28,29]. Indeed, the true function of the BG eludes any single abstract model [30,31]. However, by exploring sensory attenuation through the lens of PD, we begin to see that a reduced ability to filter environmental signals based on task-relevance creates a more complex landscape for the extraction of salient signals, just as such signals might be more beneficial to enhance movement and perception.

Finally, we examine current exercise rehabilitation techniques for further support of this notion, and consider how the most effective techniques might be working within this framework to place increasing demands on the processing capacity of depleted neurons in the BG rather than by bypassing them, and hence provide only a short-term solution. Implications for rehabilitation design are considered, pointing to sensory attenuation paradigms of a particular design to help monitor changes in important sensorimotor processing throughout an exercise programme.

## 2. Sensory Attenuation and Task-Relevance

### 2.1. Recent and Relevant Findings

Sensory attenuation usually describes the phenomenon whereby sensory input elicited by self-generated actions is reduced compared to sensory input generated externally. Anecdotally, the inability to tickle oneself has captured this phenomenon well. Elsewhere a force-matching task has been used to demonstrate that we appear to experience external forces as more intense than self-generated equivalents [32,33,34]. Sensory attenuation can also be demonstrated using electrophysiological and brain imaging techniques whereby self-generated versus externally provided cues result in attenuation of somatosensory, auditory or visual evoked potentials [35,36,37]. Sensory attenuation has proved an interesting and robust phenomenon that appears to index selective information processing within multiple modalities. In addition to being used to explain why self-tickle is ineffective [38,39], sensory attenuation has been demonstrated when simply observing actions [40,41].

Some researchers have distinguished between the attenuation of sensory evoked potentials (SEPs) at the cortical level and behavioural outcomes that indicate altered perception of a stimulus [35,42]. There is good reason for acknowledging the neurophysiological responses and the behavioural measures as the two do not always present harmoniously. For instance, in healthy older adults, sensory attenuation appears to increase with age when measured with a force-matching task [43], but the picture is a little more complex when investigating the neurophysiological data [44]. Furthermore, behavioural measures indicated equally good distinction of smooth and rough surfaces in two conditions: active touch, where the participant moves their finger across the surface; and dynamic passive touch, where the participant’s finger remains still while the surface is moved. However, fMRI analysis revealed distinct brain activation patterns for the two touch conditions [45]. Across different measures and studies, the common pattern is that identical stimuli have the potential to elicit different, context-dependent responses.

Sensory attenuation appears to be reduced in PD, displaying diminished differences between the intensity of self-generated and externally generated sensations. When comparing sensory evoked potentials from electrical stimulation of the thumb during movement and rest, patients with Parkinson’s disease who were off medication displayed reduced attenuation of the movement-initiated stimuli compared to healthy controls [35,42]. Furthermore, in the same studies, dopaminergic medication restored sensory attenuation in participants with PD. In a PD-ON group, Wolpe and colleagues [34] found that the amount of sensory attenuation was negatively related to motor symptom severity, but positively related to dopamine dose in a force-matching task. PD symptoms are of course a problematic confound when making assessments in movement-based paradigms such as the force-matching task, especially in the OFF-state, but the link between higher dopamine dose equivalent and increased sensory attenuation reinforces the connection to dopamine and its alteration in PD. In a speech task, people with PD also demonstrate reduced attenuation of auditory evoked activity 100 ms after sound onset when the participant speaks as compared to when the sound is externally produced [46].

### 2.2. Reconsidering Sensory Attenuation Theory

Prominent theories of motor control emphasise the role of predicted sensory consequences in sensory attenuation. It is believed that we construct an internal model of the world around us which is built-up through experience, and which allows us to make predictions about the results of our movements. In optimal control theory, an efference copy of a motor command is used to predict its sensory consequence to overcome sensory delays, cancelling out self-generated feedback to better detect sensory information in the environment [47]. In active inference, which subsumes predictive processing theory, the prediction itself acts as a motor command and descends further down the neural hierarchy. It is compared to the current position of the body, which gives rise to a prediction error. The overall goal is to reduce prediction error, and therefore this prediction error can either travel back up to inform higher centres of the lack of movement and update the internal model, or the prediction error is attenuated and fulfilled by reflex arcs bringing about the movement [48]. Perhaps confusingly, whilst sensory attenuation has previously been used to describe the resultant phenomenon of perceived intensity for self-generated sensations, here it describes the halting of an ascending prediction error. This halted ascension is enabled by lowering the precision of the prediction error.

Both theories utilise a Bayesian-like framework, where weighting of prior and current evidence is altered based on precision. Using prior knowledge provides a way to estimate and better navigate an environment [49]. Moving in an uncertain environment, such as playing sport at dusk, increases the reliance on prior information, and the Bayesian framework specifies how we can optimally combine multiple sources of information to better estimate an uncertain event [50]. Learning a new skill or navigating a new environment naturally involves novel unknown information, but useful information can be better extracted using predictive signals formed in a Bayesian manner from previous experience [51].

While the optimal control and active inference theories similarly predict a wide array of empirical findings, they do differ in the way that sensory attenuation during movement is described. In optimal control theory, sensory attenuation is a result of accurately predicting the sensory consequences of movement. The predictions are made with the efference copy of the motor command, providing an informative internal signal [52]. This underpins various benefits such as overcoming noise and delays in the sensorimotor system, and heightens detection of unpredicted and potentially useful/dangerous stimuli. In active inference, sensory attenuation is not just a beneficial by-product of movement, but essential in bringing it about [48]. Here sensory attenuation refers to the down-weighting of the precision of sensory signals to facilitate the fulfilment of predictions by reflex arcs. The reduced precision of a sensory signal means a reduced perception of its sensation at higher levels during movement onset; or, put another way, reduced sensory attenuation.

Evidence from studies of people with schizophrenia suggests sensory attenuation is altered across different modalities [53], and so measures may capture some phenomenon resulting from the fundamental way our brains engage in perception [54]. However, the presence of non-motor contributors to sensory attenuation [55] challenges optimal control theory which ultimately only describes a perception dependent on action. It has been maintained that self-generated sensations are attenuated, making way for externally-generated sensations to be facilitated [56] but this neat distinction does not sufficiently explain the specificity of selective sensory modulation amongst self-generated signals based on factors such as spatial location [57] or task-relevance [55]. And active inference, which places gating of sensory afference at an inseparable level from movement does not comfortably explain why sensation can often be enhanced by movement (e.g., [45,55]), though predictive processing more generally does make room for non-motor influences on perception [54,58].

Action, even when including imagined and observed action, is not the only factor that modifies perception. In a cleverly designed experiment, Heins and colleagues [40] trained participants in hurdling and tap dancing—two complex movements that generate sounds as a consequence of foot contact with the floor. After an extended period of training, participants watched point-light videos of themselves during an fMRI recording, being tasked with rating the subjective quality of their performance. Scrambled video and audio were used to gauge sensitivity to errors. More sensory attenuation, as indicated by reduced activation of the auditory cortex and more reactive ratings to sounds when scrambled, occurred in the tap-dancing condition. This was argued to be due to the different relationships that sound posed to the performance of the different tasks. The authors argue the importance of sounds elicited by tap-dancing is greater to task performance and are therefore goal-related, unlike the by-product sounds of hurdling. Whilst active modulation of sensory input likely occurred in both conditions, evidence of stronger modulation for tap-dancing supports the notion that factors other than self-generation mediate sensory attenuation.

Reduced neural responses in the ventral visual stream to images based on learned regularities—that is, only the expectedness of the image was manipulated—support the view that sensory input is modulated by non-motor factors also [59,60,61]. There has also been criticism of efference copy models for characterising motor commands as context-independent [62] with studies demonstrating motor cortex excitability changes with sensory stimulation [63,64]. Furthermore, self-generation can actually heighten as well as attenuate neural responses to sensory stimuli [55,65,66], which also challenges active inference as a model to explain all aspects of sensory attenuation. Whilst predictive processing accounts are flexible enough to incorporate various signals from the current context, a “generalised and multi-modal suppression of sensory input from the effector to enable movement” [67] does not lend itself to the differential attenuation or augmentation of sensory gain, nor do models arguing for inflexible sharpening effects from movement [68].

### 2.3. Scope of the Dominant Theories

While the active inference and optimal control models are useful, and appear anatomically viable (e.g., [69,70]), they so-far lack a solid specification in regards to the neural substrates involved in sensory attenuation itself. Rather, the behaviour consequence of sensory attenuation has been used to explain certain PD symptoms, centred on a global decrease in sensory precision of internal predictive signals which increases reliance on external sensory signals [34,48]. This explains how increasing the salience of external cues—such as lines on the floor to improve gait [12,13]—are needed to overcome movement deficits. Such approaches fail to have lasting effects though [13], and while reduced sensory attenuation in PD [34,35,42] indicates a deficit in successfully modulating sensory input, it is not clear, without further work at least, how this principle could inform therapeutic approaches. As we discuss in the final section, such theories do not entirely explain the rehabilitation effects of all therapies.

Such models may be limited in scope but are still useful in describing a mechanism that explains motor-related attenuation that need not be constrained to a single neural circuit. As far back as 1964, Giblin [71] reported reduced SEPs during voluntary movement and the gating of sensory afferent signals during movement is well documented (for a review, see [55]). The presence of such gating does not mean worsened perception during movement though, as many tasks have since shown. The classic definition describes sensory attenuation as resulting only as a consequence of self-generation, emphasising a suppression effect, which is itself limiting. Across the literature in the sensory attenuation field though, what the tasks do is measure how sensory stimuli are being modulated by organisms.

### 2.4. Factors Affecting Sensory Attenuation

Sensory attenuation measures the distinct context-dependent perception of two identical stimuli and, in doing so, captures not only our internal model of prediction, but also the salience and task relevance ascribed to each sensory input. As sensory attenuation is diminished in PD, this raises the question of which aspect of this multi-faceted process is affected by BG dysfunction: from sensorimotor integration, to salience attribution, through to predicting sensory consequences. Intriguingly, while sensory attenuation is reduced in PD, it appears to steadily increase with healthy ageing [43]. The reason for this remains unclear, but it shows that sensory attenuation is both sensitive enough to track change over time, and also potentially alterable: might it be possible to alter the sensorimotor processes captured in a sensory attenuation paradigm?

What factors do affect sensory attenuation, and what insights might they offer? The feeling of body ownership has been investigated using the rubber hand illusion where a participant is led to believe a fake hand belongs to them. Body ownership was shown to have a sensory attenuation effect, whereby somatosensory stimulation triggered by the fake hand was experienced comparably to stimulation triggered by the participant’s own hand, both of which were experienced less intensely than a standard externally-generated condition [72]. Ehrsson et al. [73] argues body ownership arises from multi-sensory signals from the body occurring synchronously. Likewise, Kilteni and Ehrsson [74] found that sensory attenuation was diminished in a self-generated force-match when the hands were held apart from one other, demonstrating the importance of congruence between visuospatial context and action. Sense of agency, while strongly connected to body ownership, arises from a movement feeling like it has been controlled by the self [72]. Sensory attenuation is often assumed to give rise to sense of agency; that is, processing self-generated stimuli differently to externally-generated stimuli contributes to the sense of agency [74]. Indeed, sense of agency and sensory attenuation are both seen to be altered in schizophrenia [75,76].

Of course, movement and sensation are intrinsically linked, and the context-dependent motor symptoms in PD support this notion: freezing-of-gait can be exacerbated by changing door-frame width [77], tactile triggers can help overcome akinesia [78], and visual and auditory cues can enhance gait [11]. Sensory attenuation experiments tend to focus on the attenuation of sensation due to movement, but other forms of perception can also be heightened as a result of movement. For instance, the execution of actions can benefit visual perception [79], and movement training, which results in improved action fluency and enhanced subsequent visual discrimination [80]. Sherwin and Sajda [81] found that expert musicians are better than novices at detecting anomalous sounds when listening to music; they recorded cortical activity corresponding to the playing hand giving rise to the possibility that imagined movement that mimicked the music enhanced auditory perception. Evidence for the dependence of perception on action (in addition to the descriptions of motor symptoms in PD being dependent on perception) support codependent and bidirectional links [80,82,83].

It might be logical, given the above, to assume that a deficit in sensation such as body awareness would be a crucial factor affecting movement in PD [78]. However, a deficit in proprioception is not itself enough to explain the motor symptoms of PD. Kammers et al. [84] found motor control to be unaffected when using the rubber hand illusion to create false information about the hand position. Here, it is possible movements were far from complex, and accurate proprioception may have been restored the moment movement was initiated. However, perception may arise from different levels of the neuraxis, whereby a sense of agency may be experienced at a conscious level while action adjustments occur automatically [75,85]. This is indeed consistent with the notion of low-level attenuation of sensory precision to fulfil actions described in active inference, while other predictive processes can still influence perception in higher regions [48,85]. In fact, predictive processing accounts might even explain both sense of ownership and sense of agency, whereby the former arises from reducing prediction error by updating the internal model (perceptual inference), and the latter arises from reducing prediction error with action (active inference) [86]. So, while body awareness can interact with sensory attenuation, movement need not invariably be disrupted by disturbances occurring in parallel that involve separate regions [87].

Finally, Redgrave et al. [88] distinguish between forms of motor control enacted by the BG that support the idea of layered control, maintaining the notion of bidirectional links between movement and sensation. It was argued that the BG loops are involved in distinct functions of goal-directed and habitual motor control, of which the latter is most affected in PD. Goal-directed control describes the conscious, cognitive control of movement, which might then be more easily disrupted when one’s attention is diverted. Habitual control describes the lower level, more inflexible, stimulus-response movements. This distinction is important in explaining why movement in some contexts, often more demanding ones, is worsened in PD. Goal-directed behaviour should not be confined to describing only that performed under conscious, attentional control; movement can arise from subcortical circuits to achieve a goal, without the movement being controlled cognitively. Rather, there is surely the capacity for a goal encoded at a cortical level to have the general effect of filtering the environment for fast, responsive movements to then achieve (e.g., “walk up the stairs”), in addition to a specific cognitive control (e.g., “move my leg”) [28,29]. It is the effect of this concept of sensory filtering by goal—or task—that we now examine.

### 2.5. Task-Relevance in Sensory Attenuation

**Figure 1:**
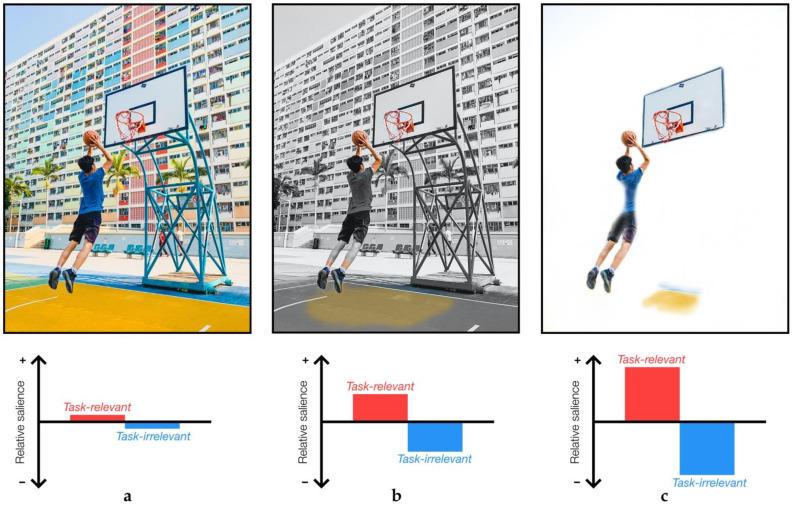
Visualising the effects of normal sensory attenuation when attempting to score in basketball, with schematic plots of the relative salience of task-relevant and task-irrelevant features: (**a**) the full, unfiltered scene is full of information, with little difference between relevant and irrelevant features; (**b**) but we are able to selectively filter information relevant to the task such as the ball, the basket, sense of the body in space relative to the ground and backboard; (**c**) salience of important stimuli may then be further enhanced through the use of contextual information; for example, using the proximity of the ball to the basket. The distance to the floor is not immediately as important, but will be once the ball has been released and the player lands.

Often overlooked in sensory attenuation studies are the effects of task demands on sensory processing. It might even seem obvious that task demands affect how we interact with our environment; the need to filter through a vastly complex and dynamic environment is crucial for an organism’s survival, and it is often considered in ecological psychology that we have evolved to do this in a task-relevant manner [89,90]. Proponents of the constraints-based approach in sports coaching strongly emphasise the importance of the three interacting elements of task, environment and organism [82] (Figure 1). However, sensory attenuation experiments largely only consider organism and environment interactions, and explain changes in sensory processing in terms relating to organism-centred factors such as movement, expectation and attention.

Task conditions affect our responses to stimuli. Staines and colleagues [91] measured responses in the human brain using fMRI, finding that during simultaneous tactile stimulation to both hands, only task-relevant stimulation increased activity in contralateral primary somatosensory cortex. In a more complex task, Riley and colleagues [92] demonstrated improved postural sway when participants were instructed to pinch and stabilise a curtain as opposed to simply making contact with it, indicating altered behavioural use of a similar tactile input when it becomes more important for the task at hand. Task complexity itself can affect the neural response to stimuli; Reiser and colleagues [93] used a mobile EEG set-up to show that auditory evoked potentials during an oddball paradigm, as well as performance of the task, were altered when participants walked an obstacle course as compared to when they walked without obstacles. Though a small motor response was required for target sounds, it does suggest that there is competition for attentional resources in a cognitive-motor dual-task paradigm, and that more complex motor tasks may depend more on such task-relevant filtering, especially in real-world environments.

**Figure 2:**
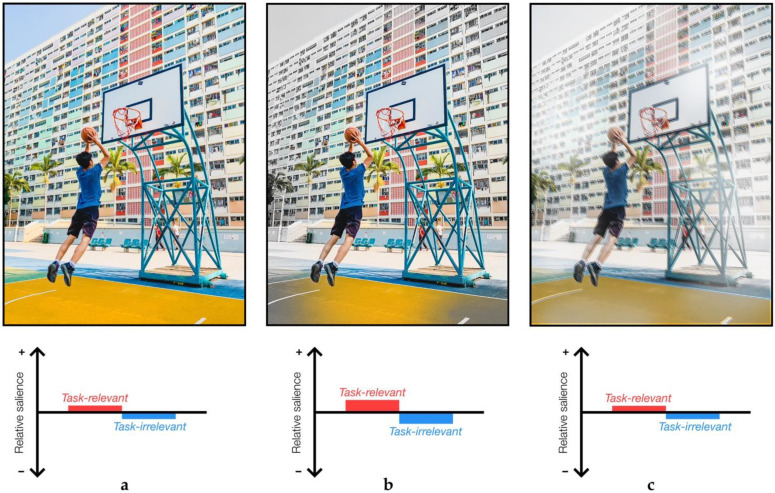
Visualising pathological sensory attenuation when attempting to score in basketball with deficits in task-relevant filtering and the utilisation of contextual information, with schematic plots of the relative salience of features important for the task: (**a**) the full, unfiltered scene is full of information; (**b**) task-relevant filtering is only partially effective, failing to filter out information irrelevant to the task and leaving little difference between task-relevant and task-irrelevant information; (**c**) this makes it harder to use contextual information to further enhance sensation, and irrelevant information remains similarly salient. The illustration made might also be considered with other examples, from everyday tasks such as the oft-considered ‘picking up a mug to drink’ example, to dance moves and other sporting scenarios.

The force-matching paradigm reveals the use of distinct contextual information in perceiving and responding to an identical target stimulus. The task in both the direct matching condition and the indirect matching condition is to match a target force, so the objective remains consistent. It has been suggested that the direct condition benefits from predictive mechanisms more so than the unusual indirect condition where the hand generates the force with a joystick or slider [32]. Interestingly, people with schizophrenia have been suggested to display increased weighting of internal predictions [94,95], whereas internal predictive signals are of reduced precision in PD [34,48], yet both appear to show less force-overcompensation in the force-matching task compared to healthy controls [34,53]. The key pattern here then is that two stimuli in two distinct contexts are perceived less differently in a disease state—that is, they converge—and this difference results from contextual information once task has been controlled for, pointing to compounding effects on sensory processing (Figure 1 and Figure 2).

Further to this point, Bolton and Staines [43,96] found reduced task-relevant modulation of sensory input during a tactile discrimination task in older adults compared to young adults. However, force-overcompensation—the sensory attenuation index in a force-matching task—was found to increase with age. This supports the notion of the force-matching task capturing the ability to utilise contextual information, such as hand position, to perceive a target stimulus and guide movement.

Perhaps this ability to utilise a contextual cue is captured by pre-pulse inhibition (PPI) and temporal discrimination tasks. In PPI paradigms, people with PD show reduced gating of auditory stimuli whereby a preceding stimulus normally attenuates the response of a subsequent stimulus when they are presented at a higher frequency [97]. Somatosensory temporal discrimination threshold (STDT) tests have also revealed people with PD have higher thresholds when discriminating between two tactile stimuli presented in close succession [98]. Researchers have pointed to a role of the BG in timekeeping operations [99,100]. Perhaps in the absence of explicit time information, the BG utilise disparate signals from across the brain to “stand-in” [101], form a “consensus” [102], and modulate a response accordingly. Indeed, the striatal networks appear to outperform the prefrontal cortex in timekeeping [103]. This points to the capacity to integrate useful contextual information for optimal behaviour and, in PD, could underpin performance deficits in PPI and STDT paradigms.

## 3. Neural Substrate

### 3.1. Reconciling Discrepancies in Studies Investigating the Neural Substrates of Sensory Attenuation

With the BG heavily associated with sensorimotor control, it is no surprise that sensory attenuation is affected in both PD and schizophrenia [53,76], both of which are disorders of the BG. It has even been postulated that fronto-striatal circuits may help explain changes of sensory attenuation in healthy older adults [34,43]. However, cortical regions such as the supplementary motor area have also been implicated [104], as has the cerebellum [56,105], and the thalamus [91]. Initially this may point to a non-specific cross-modal sensory attenuation phenomenon that describes a generalisable principle of perception and, while this may hold some truth, there is an important role for the BG in actively modulating sensory input. In fact, by examining the available literature concerning the neural basis of sensory attenuation, there appears to be a striking discrepancy that relies on the variety of tasks being used.

The findings from fMRI studies investigating sensory attenuation can broadly be split into those that show involvement of the cerebellum, those that demonstrate BG involvement, and those that show both. Pointing to cerebellar involvement, Kilteni and Ehrsson [105] demonstrated increased activity of the cerebellum and secondary somatosensory cortex in healthy young adults for self-generated sensations compared to externally generated sensations, which replicates some of the findings of Blakemore and colleagues [56]. Likewise, Boehme and colleagues [106] compared people with ADHD and neurotypical controls in a self- vs. other-touch task, finding not only a difference between groups in the primary somatosensory cortex, but also an increased BOLD signal of the cerebellum and prefrontal cortex amongst other areas when the experimenter administered touch compared to self-touch.

Capturing a combination of both BG and cerebellum involvement, a comparison of people with schizophrenia and healthy controls [53] demonstrated that the cerebellum was active in both groups during a modified force-matching task. They speculated that the cerebellum seemed to be performing a comparator function. However, they also found increased activation of the caudate for healthy controls when sensation was synchronous with movement, and increased cerebellar activation when sensation was delayed (along with other differences across condition and group). Simões-Franklin and colleagues [45] compared active, passive and dynamic passive touch for rough surface detection, reporting greater activation of the cerebellum and lentiform nucleus for the active condition compared to the passive conditions.

Supporting BG involvement, Leube et al. [107] compared people with schizophrenia to healthy controls during a task comparing visual consequences of self-generated actions in conditions of delay and no delay. They found reduced activation of the putamen in patients which accompanied a reduced ability to discriminate the delayed action consequences. Nonetheless, to add to the assortment of findings, Ackerley and colleagues [108] reported altered activations across a range of sensory areas elicited by a paint brush on the arm under self-touch and passive touch conditions, but not the BG or the cerebellum.

This somewhat mixed set of findings may best be explained by the variety of tasks used. The studies attempting to recreate the force-matching tasks have needed to heavily modify the task for use in (usually) an MRI scanner, and the constraints of such neuroimaging techniques make altered paradigms necessary. Movement may also be controlled differently in such paradigms, and is often triggered by a very simple visual cue, without the clear task goal in the behaviour-only paradigms where matching a previous force in a more complex environment demands active selective filtering using memory and task information in the absence of simple visual cues.

The self- vs. other-touch paradigms appear more consistent in design [56,106,108] with some commonalities in findings such as modulation of primary somatosensory cortex, but also differences. Movement is still instructed with an on-screen cue which might not be so problematic in this case, but there is no clear task goal and therefore no monitoring of performance or control of attention which may affect perception as well as the way movement is being initiated [108].

Perhaps the most informative designs are those which embrace the reality of a different set up and use videos of participants’ movements in the scanner, and/or manipulate visual or auditory information to alter its predictability [40,107]. This builds on the assumption that there is an attenuation effect from observing action as well as performing it [41].

Heins and colleagues [40] only compared conditions where a strong sensory attenuation effect was expected, but Leube et al. [107] compared healthy controls and people with schizophrenia. Participants opened and closed their hands which was filmed and played back for them to see in the scanner with no delay or with small delays to create congruent and incongruent action consequences. Not dissimilar to Heins et al. [40] where participants rated movement quality which worsened with scrambled videos, participants were tasked with detecting the synchrony of the video with their movements [107]. Those with schizophrenia were more likely to incorrectly perceive movements as asynchronous, and sometimes also incorrectly perceived asynchronous movements as synchronous. Such error detection paradigms used in these two studies index the ability to distinguish self from externally generated stimuli captured in sensory attenuation measures, with better detection thresholds during related movement [57,109,110]. Activation of putamen and thalamus was reduced in patients compared to controls during this error detection task during distinct delay and no-delay conditions [107].

There are of course challenges in collecting this type of data in those with movement disorders, but considerations should be made for the different types of motor control that are likely being invoked in these designs. Externally triggered movements, such as when a finger-tap is elicited by an on-screen stimulus [105], have been strongly associated with cerebellar circuits, whereas internally generated movement—that is, movement elicited and guided more so by memory signals—are associated with BG circuits [101,111,112]. If the BG are predominantly involved in memory-guided movement, and filter information for action in a task-relevant manner [29], then investigation of sensory attenuation in BG disorders should consider task design, including sensory cues, carefully. Cerebellar circuits are also altered in PD though, which is thought to be pathophysiological rather than compensatory [111]. Perhaps these systems might work in a complementary fashion whereby global task-dependent filtering decomposes a complex environment into smaller and simple components that can be responded to with other more appropriate systems such as the cerebellum [113]. A future direction for research in sensory attenuation might be to compare attenuation during different forms of motor control for further insight into its neural underpinnings.

### 3.2. The BG through a Sensory Lens: Task-Relevant Signaling

Movement can vary across environments, perhaps radically demonstrated by athletes in the phenomenon of choking in sport [114]. Motor symptoms in PD are also responsive to sensory environment and task demand, which are exacerbated in more challenging environments [77,78,115,116,117] but alleviated when aided by sensory cues that help navigate an environment [10,12,13,14]. This leaves a narrow set of often impractical environments where free-flowing movement is possible. Importantly though, this demonstrates that PD is not purely a kinetic disorder, and rather that the BG has a sensory processing function that heavily impacts movement [26,27,29,118].

Before movement is even considered, the BG appear influential in task-relevant sensory filtering to achieve behavioural goals. Nakajima and colleagues [28] describe a need for animals to filter relevant stimuli through sensory noise. It has been shown that the need to engage sensory filtering can depend on the amount of sensory noise and also behavioural goals [119]. Goal-related movement has often been functionally linked to the BG [30], but such behaviourally-relevant sensory filtering has also been demonstrated in non-motor aspects of behaviour. McNab and Klingberg [120] demonstrated BG involvement in filtering visual stimuli, and suggested that working memory capacity may be related to how well (ir)relevant information is filtered, finding that increased globus pallidus activity correlated with increased working memory capacity. It has also been demonstrated that memory for deep-encoded words is impaired in PD compared to shallow-encoded words, with higher beta oscillations (an indicator of reduced novel processing) during deep-semantic processing, suggesting a difference in the initial encoding phase [24]. In a recent study, evidence was found for a pathway between the prefrontal cortex and the BG which mediates top-down filtering of irrelevant stimuli based on task demand and not movement [28].

Considering a task-dependent sensory processing role appears fruitful in motor control studies too. Neuronal recordings have shown that the BG respond to sensory stimulation more prominently when they are relevant for upcoming motor control [26]. This may provide a mechanism to explain how BG lesions mostly affect automatic movements that require sensory guidance [26]. Schneider [118] meanwhile argues for a transient and adaptable system which respond differently to stimulation during different tasks. Schneider et al. [121] found that in cats, neurons in the entopeduncular nucleus (homologous to the internal segment of the globus pallidus in humans) and the caudate nucleus do not respond to facial stimulation or jaw movement, but those cells do become responsive if they are stimulated during ingestion-related jaw movements. These findings suggest a more complex and dynamic relationship to movement, and the BG system is well placed to monitor internal signals relevant to task demands with input from across the cortex [122].

Understanding the role of the BG in a sensory capacity begins to make sense of some of the context-specific motor symptoms in PD, and lays a foundation for the existence of a common mechanism to underlie the vast array of motor and non-motor symptoms in PD. BG disorders are themselves diverse, expressing a range of motor, cognitive and emotional symptoms [31,118,122]. It is worth considering then, that damage to the BG in PD affects processes that underpin environment-specific motor symptoms, in addition to non-motor symptoms. A viable alternative might be that non-motor symptoms arise from the overload of more cognitive pathways in PD which may be required to control movement due to degeneration of circuits involved in habitual (lower-level, stimulus-response) control of movement [88]. There is evidence however of both reduced storage capacity and an impaired ability to filter out irrelevant information that underlies such non-motor functions as visual working-memory [123], which hints at a more general sensory filtering function of the BG that could underpin some motor and non-motor PD symptoms.

### 3.3. Sensation to Action

How might impaired sensory filtering be important not just in enhancing movement more generally, but the initiation of actions? A possible mechanism has been demonstrated in early animal experiments. Researchers showed that electrically stimulating the lateral hypothalamus of a cat would sensitise the perioral area, so that touching the area around the mouth would cause the animal to orient toward the stimulus and open its mouth. This reflex would only work when the hypothalamus was stimulated [124], so by modulating sensory processing, movement was initiated. In an excellent review, Robbe [29] argues against the BG as an action selector. Instead, the BG continuously track sensorimotor signals, which can contribute to action production. Such a sensorimotor transformation is demonstrated in membrane potential recordings of medium spiny neurons (MSNs) in mice trained to lick a reward spout following a whisker deflection [125]. The MSNs depolarised during whisker deflection after training, and optogenetic stimulation of those neurons was able to substitute for the whisker deflection and elicit the licking action. During whisker deflection though, the MSNs were more active for successful trials compared to unsuccessful, indicating that a motor response could be elicited with a predictive sensory stimuli [29]. Interestingly, it was the MSNs in the direct pathway—the pathway that is underactive in PD—that depolarised with the whiskers and predicted a licking response [125].

### 3.4. Sensorimotor Integration

The BG are strongly positioned to monitor internal and external signals, contributing to motor responses. The generation of actions appropriate to task and environment requires integration of signals from multiple sources [29,126]. Motor symptoms in PD reveal deficits in sensorimotor integration. Consider the finding that people with PD display difficultly tracking a visual target on screen by using jaw movements to control another onscreen signal [127]. There are multiple sources of information that require monitoring and integration to successfully perform the task, both internal and external, across modalities. However visually guiding movement is often helpful or even necessary in PD, and motor deficits can be overcome with visual cues [12,13]. One explanation might be that reducing the demands on integration processes of the BG, achieved by using only vision to guide the foot to a line on the floor for example, restore functionality [128]. In support, bilateral integration from whisker stimulation by MSN was found to be diminished in dopamine-depleted mice [129]. Alternatively, visual cueing strategies might make use of alternative and more intact pathways [7,111], but it is intriguing that using visual information during movement in different ways can have opposing effects on movement quality.

With a wide range of inputs to the BG and a role integrating multimodal information for action, studying oscillatory activity across neural systems can prove useful to track complex processing dynamics. Oscillatory changes in PD have been well established, notably in the basal ganglia [130,131,132] but also in cortex [133,134]. Of particular interest, there has been a focus on the modulation of activity within the alpha/mu (8–12 Hz) and beta (13–30 Hz) frequency bands [130]. Focussing on PD, the time-course of beta has been shown to index movement kinematics [135], the severity of motor impairment [136], and cortico-muscular coupling at beta frequencies is reduced in both early and late stages of PD [133,137]. These observations reinforce arguments we have made about the role of the BG, as well as offering insight into changes in sensorimotor processing in PD. Changes in neural synchrony, by means of phase-resetting or neural entrainment, offer potential mechanisms for instigating differential responses to sensory stimuli [138]. Beta synchrony in the BG is reduced during movement, but also in response to cues that are predictive of movement [139]. Whilst it has been argued that beta synchrony indexes the likelihood for a need for a new action [131], it might more fundamentally signify the capacity for processing new information [130] which resultantly may facilitate action. Excessive beta synchrony impedes response to novel demands, creating a neural landscape unable to match the complexity of surrounding environments [122,130], harming vital task-dependent sensory filtering and sensorimotor integration processes necessary for appropriate behavioural responses (Figure 2).

## 4. Establishing Principles for Rehabilitation

How do we enhance such vital task-dependent sensory filtering and sensorimotor integration processes? Returning to the motor control theories which point to an imbalance between predictive and sensory signals, we can see how altering information in the sensory environment can affect movement. Rudimentary sensory cues often simplify and contextualise the perceived environment, which can improve motor performance, but without demanding the ability to filter and extract useful information in more complex surroundings. To surpass the malfunctioning sensorimotor integration described in optimal control and active inference models, the integrating capacity of the human brain must then also increase to negotiate a complex environment without guides. Crucially, if the BG play a role in task-relevant filtering and then also in utilising useful signals for behaviour, how do we exercise this system?

Sage and Almeida [7,8] utilised a form of exercise called PD-SAFEx where limb movements and gait exercises were performed in low-light conditions, increasing attention to proprioceptive input for guiding movement, forcibly shifting it away from visual input. The researchers found the programme improved symptom severity scores at the end of the programme and after a non-exercise washout period of 6 weeks. We propose that clear task goals, such as alternating hand to ear with every step while walking, and a requirement to utilise proprioceptive signals to guide movement without any pre-filtered cues create a demand that must be met.

Another example of a promising rehabilitation method is forced-exercise cycling [17]. An early example of this illustrates the concept well; Ridgel and colleagues [18] had participants perform tandem cycling where they had to maintain the same high cadence dictated by the other cyclist. Participants with PD improved their symptom severity scores after an 8-week training programme. A later study ruled out the possibility of this improvement simply being caused by the high output and high proprioceptive stimulation [19] and instead we suggest there is a demand on the participant to integrate their own movement with that of another cyclist, while a clear task goal is available to filter only the useful signals from the otherwise unfiltered dynamic environment.

There is a deficit in PD first of using task-relevance to filter complex environments which is mediated by the BG [28,29,89,140] and then to use this information as useful contextual cues for behaviour [24,101,102,118]. We employ the analogy of noise-cancelling headphones to consider this effect. Without cancellation it is difficult to pick out a target sound or a voice which might be needed to achieve some goal. However, once noise-cancelling is active, using relevant sounds for the task becomes much easier. We can think of unmedicated PD as a condition in which the ‘task-relevant’ noise-cancellation is turned off. This is further demonstrated by dopaminergic modulation in the force-matching task, whereby higher dopamine doses accompanied a bigger difference between two matching conditions, indicating greater utilisation of unique contexts during movement [34].

Nonnekes and colleagues [141] emphasised the inventiveness of PD patients in developing effective compensatory strategies to overcome gait deficits. Such strategies might be considered more effective because they are developed within the specific environment that the patient navigates. As the context of an event affects our response to it, rehabilitation in daily-life settings might be appealing. However, this could highlight the importance of the compatibility of a strategy, and not necessarily transfer specificity. When considering specificity, it is important to recognise not just that we are better at recalling something in the same physical space it was encoded [142], but also that internal factors such as attentional context can affect motor learning [143]. In sport, specificity considerations underline factors such as muscle fatigue, force landscapes and speed of movement, all of which form a context that may affect transfer from practice to competition [82]. Furthermore, the evidence actually points to a reduced ability in PD to make use of such contextual factors during recall [24]. This poses a challenge, but as no two contexts of a movement are ever the same, perhaps it is better to train the underlying ability to identify and utilise contextual cues and to do so in a range of interesting environments that invite exploration and the use of task-relevant filtering to draw out cues to enhance movement.

With the short-term success of simple sensory cues, there might be a temptation to bombard a patient with cues to overcome motor deficits. However, in the absence of simple cues the BG are required to incorporate other internal signals relating to task and context to make sense of a complex environment [101,102]. Finally, task will alter what signals are relevant and irrelevant, meaning a movement may look the same but still affect the brain’s response to that movement [21,91], which is ultimately the principle underpinning sensory attenuation—active modulation of sensory input. Determining sensory input in an exercise programme is not sufficient; task and environment critically alter our response to each sensory event, and therefore our experience and capacity to engage with the world as a whole.

## 5. Conclusions

We have reviewed sensory attenuation through the lens of PD with a particular interest in sport and rehabilitation. Studies investigating sensory attenuation measure the distinct responses that can emerge from two identical stimuli presented under different contexts. Differences in these responses indicate that contextual information has been utilised in active modulation of the sensory input, captured by a facilitation or suppression of the response to a stimulus. Diseased states appear to cause a convergence of responses that are more differentiable in healthy populations, indicating a reduced ability to integrate relevant sensory signals for perception or to enact appropriate movements. However, a crucial step precedes the utilisation of contextual cues, and we have considered the importance of task-relevance in both reconciling seemingly conflicting findings in sensory attenuation research and simultaneously informing on the symptoms of Parkinson’s disease. There is a dual effect of BG dysfunction in PD where a reduced ability to filter relevant signals from a complex environment overwhelms a BG system already at reduced capacity, just when contextual cues might be used to enhance movement and perception (Figure 1 and Figure 2). Sensory attenuation paradigms may capture this process and offer a useful tool to track changes elicited by effective interventions and may even form the foundations of effective rehabilitation strategies themselves. Regardless, examining the sensorimotor processes that underpin sensory attenuation through the lens of PD pave the way for establishing key principles that may be useful in guiding the design of effective rehabilitation.
